# Unveiling the Binding between the Armadillo-Repeat Domain of Plakophilin 1 and the Intrinsically Disordered Transcriptional Repressor RYBP

**DOI:** 10.3390/biom14050561

**Published:** 2024-05-07

**Authors:** Salome Araujo-Abad, Bruno Rizzuti, Miguel Vidal, Olga Abian, María Esther Fárez-Vidal, Adrian Velazquez-Campoy, Camino de Juan Romero, José L. Neira

**Affiliations:** 1 Cancer Research Group, Faculty of Engineering and Applied Sciences, Universidad de Las Américas, 170124 Quito, Ecuador; lourdes.araujo@udla.edu.ec; 2 IDIBE, Universidad Miguel Hernández, 03202 Elche, Spain; 3 CNR-NANOTEC, SS Rende (CS), Department of Physics, University of Calabria, 87036 Rende, Italy; bruno.rizzuti@cnr.it; 4 Institute of Biocomputation and Physics of Complex Systems (BIFI), Universidad de Zaragoza, 50018 Zaragoza, Spain; oabifra@unizar.es (O.A.); adrianvc@unizar.es (A.V.-C.); 5 Centro de Investigaciones Biológicas Margarita Salas (CSIC), Calle Ramiro de Maeztu, 9, 28040 Madrid, Spain; mvidal@cib.csic.es; 6 Instituto de Investigación Sanitaria Aragón (IIS Aragón), 50009 Zaragoza, Spain; 7 Centro de Investigación Biomédica en Red en el Área Temática de Enfermedades Hepáticas y Digestivas (CIBERehd), 28029 Madrid, Spain; 8 Departamento de Bioquímica y Biología Molecular y Celular, Universidad de Zaragoza, 50009 Zaragoza, Spain; 9 Departamento de Bioquímica y Biología Molecular III e Inmunología, Facultad de Medicina, Universidad de Granada, 18016 Granada, Spain; efarez@ugr.es; 10 Instituto de Investigación Biomédica IBS, Granada, Complejo Hospitalario Universitario de Granada, Universidad de Granada, 18071 Granada, Spain; 11 Unidad de Investigación, Fundación para el Fomento de la Investigación Sanitaria y Biomédica de la Comunidad Valenciana (FISABIO), Hospital General Universitario de Elche, Camí de l’Almazara 11, 03203 Elche, Spain

**Keywords:** immunofluorescence, protein–protein interactions, intrinsically disordered protein, PKP1, isothermal titration calorimetry, molecular modelling, proximity ligation assay

## Abstract

Plakophilin 1 (PKP1), a member of the p120ctn subfamily of the armadillo (ARM)-repeat-containing proteins, is an important structural component of cell–cell adhesion scaffolds although it can also be ubiquitously found in the cytoplasm and the nucleus. RYBP (RING 1A and YY1 binding protein) is a multifunctional intrinsically disordered protein (IDP) best described as a transcriptional regulator. Both proteins are involved in the development and metastasis of several types of tumors. We studied the binding of the armadillo domain of PKP1 (ARM-PKP1) with RYBP by using in cellulo methods, namely immunofluorescence (IF) and proximity ligation assay (PLA), and in vitro biophysical techniques, namely fluorescence, far-ultraviolet (far-UV) circular dichroism (CD), and isothermal titration calorimetry (ITC). We also characterized the binding of the two proteins by using in silico experiments. Our results showed that there was binding in tumor and non-tumoral cell lines. Binding in vitro between the two proteins was also monitored and found to occur with a dissociation constant in the low micromolar range (~10 μM). Finally, in silico experiments provided additional information on the possible structure of the binding complex, especially on the binding ARM-PKP1 hot-spot. Our findings suggest that RYBP might be a rescuer of the high expression of PKP1 in tumors, where it could decrease the epithelial–mesenchymal transition in some cancer cells.

## 1. Introduction

RYBP (RING1A and YY1 binding protein, UniProt number Q8N488) was first characterized as an interacting partner of the Polycomb group (PcG) protein RING1A [1] and a non-canonical component of the Polycomb Repressive Complex 1 (PRC1) [2,3,4,5]. The PcGs are transcriptional repressors involved in multicellular development and in cancer epigenetics among other functions [6,7]. Non-canonical PRC1 isoforms are specifically defined as protein complexes whose minimal functional cores are formed by the E3 ubiquitin ligase subunit RING1B (or its paralog RING1A), one of the six Polycomb group ring-finger domain (PcGF) subunits, and RYBP, or its paralog YY1-associated factor 2 (YAF2) [4,8,9], a member of the E2F family of transcription factors [10].

The presence of RYBP is crucial for the pathways involving non-canonical PRC1 isoforms because it improves the enzymatic activity of the catalytic RING1B/PcGF complex [4,11,12]. RYBP is also involved in PRC1-independent cellular pathways, and it can modulate apoptosis through (i) the regulation of the activity of proteins of the necrosis factor alpha receptor family; (ii) the stabilization of p53 by binding to the E2-ubiquitin ligase MDM2; or (iii) direct interaction with the death effector domain (DED)-containing proteins [2,13,14,15,16]. Thus, RYBP can work both as a tumor suppressor [17] and as an oncogene [2,13]. Furthermore, RYBP can act as an inhibitor protecting against the progression of distant metastases and the recurrence of the malady in colorectal cancer [18]. RYBP inhibits the progression and metastasis of lung cancer, suppressing the epidermal growth factor receptor (EGFR) signaling and the epithelial–mesenchymal transition (EMT) [19]. EMT is a key mechanism underlying cancer metastasis and invasion [20,21]; it is characterized by the loss of the epithelial phenotype and acquisition of the mesenchymal one, as well as by the loss of cell–cell polarity and adhesion. From a functional point of view, RYBP is capable of binding to ubiquitylated proteins [2,22,23]. Therefore, RYBP appears to be an example of a multi-tasking protein participating in several other protein cross-talks [24].

**Figure 1:**
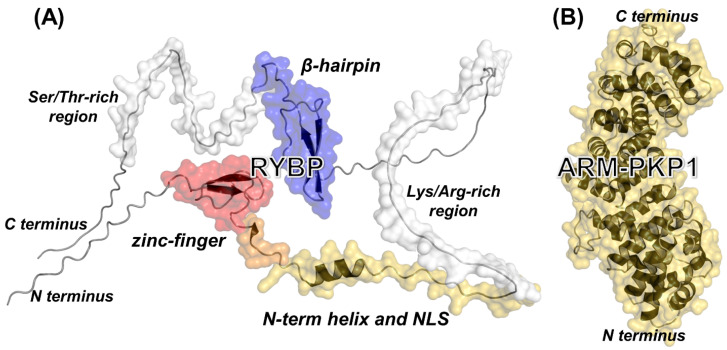
Structure of the RYBP model and ARM-PKP1. (**A**) Structure of the RYBP model as predicted by AlphaFold [55]. Most ordered regions are the following: fragment 20–55 (red and orange), including the zinc-finger domain at residues 23–47; fragment 50–85 (orange and yellow), encompassing the N-term helix at residues 59–69 and the NLS of RYBP; and fragment 145–180 (blue), including the C-terminal β-hairpin at residues 165–175. Other protein regions are unfolded, including one rich in Lys/Arg residues amidst residues 77–119 (partly included in fragment 50–85) and another rich in Ser/Thr residues amidst residues 181–214. (**B**) Structure of the ARM-PKP1 (from the PDB entry 1XM9, containing residues 244–700 of the whole protein).

From a structural point of view, RYBP is a 228-residue-long, highly basic intrinsically disordered protein (IDP) that binds to DNA [2,25]. As shown in Figure 1A, in the RYBP model, the primary structure includes a conserved zinc-finger motif at the N terminus, which is folded and contains the ubiquitin-binding domain [22,26] followed by a so-called ‘N-term’ helix [27] ending with the nuclear location signal (NLS) that allows for its nuclear translocation [28] and, further ahead, by a β-hairpin motif. The rest of the structure of the RYBP model is unfolded, including a polypeptide region rich in Lys and Arg residues and a C-terminal region with a high percentage of Ser and Thr residues. Like other IDPs, the RYBP model does lack a unique stable tertiary conformation, resulting in a high structural flexibility and paving the way to interact with several macromolecules [29,30,31,32] as described above, including also citrullinating enzymes [33].

Plakophilins (PKPs) belong to the armadillo (ARM)-repeat-containing protein family, and they are located at cell–cell junctions, particularly in desmosomal structures [34]. PKPs are ubiquitously found in the cytoplasm and nucleus in several types of cells [35,36], where they intervene in signaling networks within distinct cellular compartments. There are four PKP-family members: PKP1, PKP2, PKP3, and PKP4 [37]. PKP1 (UniProt number Q13835) acts as a modulator of mRNA translation and post-transcriptional gene expression [34,38,39], and it is more largely expressed in the supra-basal layers of stratified and complex epithelia [40,41,42]. This protein has been proposed as both a valuable diagnostic biomarker as well as a potential therapeutic target in lung squamous cell carcinoma [38,39,43,44,45]. In fact, high levels of PKP1 have been detected in patients with breast and lung cancers [46], and such a high expression has been correlated with a lower survival rate and a worse disease progression in patients. PKP1 promotes cancer cell survival and metastasis via cluster formation in the circulatory system [46] and, as it happens with RYBP, it has been reported to be both an oncogene and a tumor suppressor [38,47]. It seems that the different distribution patterns of the PKPs in carcinomas can be related to the differentiation-specific expression within normal epithelia, as well as to the cell type involved.

The structure of the ARM-repeat domain of PKP1 (ARM-PKP1) has been solved via X-ray (Figure 1B); it contains nine ARM motifs and includes a large basic patch serving as a binding site region for most of its partners [48,49,50]. We have previously shown that isolated ARM-PKP1 is a monomer in solution, and it has a low conformational stability [51]. Furthermore, it interacts with the sterile alpha motif (SAM) of p73 [52] and with NUPR1, an IDP intervening in the development of pancreatic cancer [53]. Since PKP1 and RYBP (i) are involved in the development of several types of cancers, (ii) can both be oncogenes and tumor suppressors, and (iii) mutually interact with the citrullinating enzyme PADI4 [33,54], we hypothesized that RYBP could also be capable of binding to ARM-PKP1.

In this study, we provided evidence for the binding between ARM-PKP1 and RYBP by using in cellulo, in vitro, and in silico techniques. All these methods provided evidence for the binding between the two proteins, and the in cellulo findings indicate that association occurred in the cytoplasm and in the nucleus but was cell-line-dependent. The dissociation constant of the complex, *K*_d_, was moderate (~10 µM), as measured via ITC and fluorescence titrations. Our findings could be of importance to understand how cancer development or even the EMT mechanism can be regulated in the cell; in fact, we hypothesize that RYBP could act as a rescuer in the presence of a large amount of PKP1 in some types of tumor cells.

## 2. Materials and Methods

### 2.1. Materials

Imidazole, Trizma base and acid, DNase, SIGMAFAST protease tablets, NaCl, Ni^2+^-resin, anti-PKP1 antibody, DAPI (4′,6-diamidino-2-phenylindole), paraformaldehyde (PFA), and ultra-pure dioxane were obtained from Sigma (Madrid, Spain). Ampicillin, kanamycin, and isopropyl-β-D-1-thiogalactopyranoside were obtained from Apollo Scientific (Stockport, UK). Dialysis tubing with a molecular weight cut-off of 3500 Da, Triton X-100, TCEP (tris(2-carboxyethyl)phosphine) and the SDS protein marker (PAGEmark Tricolor) were obtained from VWR (Barcelona, Spain). Amicon centrifugal devices with a molecular weight cut-off of 30 kDa were obtained from Millipore (Barcelona, Spain). The rest of the materials were of analytical grade. Water was deionized and purified on a Millipore system.

### 2.2. Protein Expression and Purification

RYBP and ARM-PKP1 were purified as previously described [25,51]. Protein concentrations were determined through UV absorbance, employing an extinction coefficient at 280 nm estimated from the numbers of tyrosines and tryptophans in each protein [56].

### 2.3. Cell Lines

Breast adenocarcinoma cells, MDA-MB-231(catalog number 92020424) and human fetal lung fibroblast cells, MRC-5 (catalog number 05072101) were purchased from Sigma-Aldrich (Madrid, Spain). The MDA-MB-231 cell line was cultured in Dulbecco’s Modified Eagle’s Medium: High Glucose (DEMEM-HG) (Biowest, MO, USA) and supplemented with 10% (*v*/*v*) heat-inactivated fetal bovine serum (FBS) (Capricorn Scientific, Ebsdorfergrund, Germany) and 1% (*v*/*v*) penicillin/streptomycin mixture (Biowest, MO, USA). MRC-5 cells were cultured in Minimum Essential Media (MEM) (Biowest, MO, USA) enriched with 10% FBS, 1% (*v*/*v*) penicillin/streptomycin mixture, and 2 mM L-glutamine. Both cell cultures were incubated at 37 °C in a humidified 5% CO_2_ atmosphere.

### 2.4. Immunofluorescence (IF)

A total of 35,000 cells of MRC-5 or MDA-MB-231 cell lines were seeded into twenty-four-well plates on coverslips. After 24 h, they were fixed with PFA at 4% concentration and blocked with FBS/PBS (1×) (50 μL/mL). Next, cells were incubated with anti-PKP1 (1:100, mouse; Invitrogen, Barcelona, Spain) and anti-RYBP (1:100, rabbit; homemade) antibodies [1]. After washing out the first antibody, cells were incubated with Alexa Fluor 568-labeled anti-mouse (1:500) and Alexa Fluor 488-labeled anti-rabbit (1:500) secondary antibodies (Invitrogen, Barcelona, Spain); the DAPI reagent was used to stain the nucleus. Coverslips were mounted in Prolong™ Gold Antifade Reagent (Invitrogen, Barcelona, Spain) and analyzed using a confocal microscope LSM900 with Airyscan 2 (Carl Zeiss, Oberkochen, Germany) at 63× magnification.

### 2.5. Proximity Ligation Assay (PLA)

A total of 35,000 cells of MRC-5 and MDA-MB-231 cell-lines were seeded in twenty-four-well plates on coverslips. After 24 h, cells were washed in PBS (1×), fixed with PFA 4%, washed again, permeabilized in PBS (1×) with 0.2% Triton X-100, and blocked with blocking solution for 1 h at 37 °C before immune staining with Duolink by using PLA Technology (Merck, Madrid, Spain), following the manufacturer’s protocol. Anti-PKP1 and anti-RYBP primary antibodies were used. Then, slides were processed for in situ PLA by using, sequentially, the Duolink In Situ PLA Probe Anti-Mouse MINUS, Duolink In Situ PLA Probe Anti-Rabbit PLUS, and Duolink In Situ Detection Reagents Red (Merck, Madrid, Spain). In such experiments, red fluorescence spots correspond to the PLA-positive signal, and they indicate that the two proteins are bound, forming a protein complex, whereas the blue fluorescence signals correspond to nuclei (DAPI staining). Both negative and positive control experiments were performed, the former by omitting one of the primary antibodies. As in the case of IF experiments, image acquisition was carried out by using a confocal microscope LSM900 with Airyscan 2 (Carl Zeiss, Oberkochen, Germany) at 63× magnification.

### 2.6. Fluorescence

#### 2.6.1. Steady-State Fluorescence

A Cary Varian spectrofluorometer (Agilent, Santa Clara, CA, USA), interfaced with a Peltier unit, was used to collect fluorescence spectra at 25 °C via excitation at either 280 or 295 nm; slit widths were 5 nm. The other experimental parameters have been described elsewhere [57]. Appropriate blank corrections were made in all spectra. Following the standard protocols used in our laboratories, the samples were prepared the day before and left overnight at 5 °C; before experiments, samples were left for 1 h at 25 °C. A 1 cm path length quartz cell (Hellma, Kruibeke, Belgium) was used. Concentration of RYBP was 20 μM and that of ARM-PKP1 was 2 µM. Experiments were performed in 20 mM Tris buffer (pH 7.5), 5 mM TCEP, and 150 mM NaCl in triplicates with newly prepared samples. Variations in results among the experiments were lower than 5%.

#### 2.6.2. Steady-State Fluorescence

For the titration of ARM-PKP1 with RYBP, increasing amounts of monomeric ARM-PKP1 species, in the concentration range 0–20 µM (final concentration in the solution), were added to a solution with a fixed concentration of RYBP (4.6 µM, final concentration). Experiments were carried out in the buffer described above with the same experimental set-up. Spectra were corrected for inner-filter effects [58]. The titration was repeated three times, using new samples; in all cases, the variations were lower than 10%.

The dissociation constant of the complex, *K*_d_, was calculated by fitting the binding isotherm obtained by plotting the observed fluorescence change as a function of ARM-PKP1 concentration to a general binding model, explicitly considering ligand depletion [59,60]:

(1) $$F=F_{0}+\frac{∆F_{max}}{2{\left[RYBP\right]}_{T}}{{(\left[ARM-PKP1\right]}_{T}+{\left[RYBP\right]}_{T}+K}_{d})-\sqrt{\left({{{(\left[ARM-PKP1\right]}_{T}+{\left[RYBP\right]}_{T}+K}_{d})}^{2}-4{\left[ARM-PKP1\right]}_{T}{\left[RYBP\right]}_{T}\right)}$$

Here, *F* is the measured fluorescence of the solution with the fixed RYBP concentration (4.6 μM), [RYBP]_T_, and a particular ARM-PKP1 one, [ARM-PKP1]_T_, after subtraction of the corresponding blank with the same concentration of ARM-PKP1; Δ*F*_max_ is the largest change in the fluorescence of ARM-PKP1 when all polypeptide molecules were forming the complex; and *F*_0_ is the fluorescence intensity when no ARM-PKP1 was added. Fitting of data to Equation (1) was carried out by using KaleidaGraph (Synergy software, Reading, PA, USA).

### 2.7. Circular Dichroism (CD)

Far-UV CD spectra were collected on a Jasco J810 spectropolarimeter (Jasco, Tokyo, Japan) with a thermostated cell holder and interfaced with a Peltier unit. The instrument was periodically calibrated with (+)-10-camphorsulfonic acid. A 0.1 cm path length quartz cell was used (Hellma, Kruibeke, Belgium). All spectra were corrected by subtracting the corresponding baseline. Concentration of each polypeptide (ARM-PKP1 or RYBP) and the buffers were the same as what were used for fluorescence experiments (Section 2.6.1). Samples were prepared the day before and left overnight at 5 °C to allow them to equilibrate. Before starting the experiments, samples were further left for 1 h at 25 °C.

Isothermal wavelength spectra of each isolated macromolecule and those of the complex were acquired as averages of 6 scans, at a scan speed of 50 nm/min, with a response time of 2 s and a band-width of 1 nm.

### 2.8. Isothermal Titration Calorimetry (ITC)

Calorimetric titrations for testing the interaction of ARM-PKP1 with RYBP were carried out in an Auto-iTC200 automated high-sensitivity calorimeter (MicroCal, Malvern-Panalytical, Malvern, UK). Experiments were performed at 25 °C in 20 mM Tris buffer (pH 7.5), 5 mM TCEP, and 150 mM NaCl. RYBP (100 μM) in the injection syringe was titrated into the ARM-PKP1 solution (10 μM) in the calorimetric cell. A series of 19 injections with 2 μL volume, 0.5 μL/s injection speed, and 150 s time spacing were programmed while maintaining a reference power of 10 μcal/s and a stirring speed of 750 rpm. Integration of the thermal power raw data was used to calculate the heat effect per injection. The interaction isotherm, which is the ligand-normalized heat effect per injection as a function of the molar ratio, was analyzed through non-linear least squares regression data analysis. In such analysis, we used a model that considers a single binding site to estimate the association constant, *K*_a_; the interaction enthalpy, Δ*H*; and the stoichiometry of binding, *n*, although, in practice, the latter usually reports the fraction of active protein in the cell/syringe. The background injection heat (usually called “dilution heat”, but reflecting any unspecific phenomenon such as solute dilution, buffer neutralization, temperature equilibration, or solution mechanical mixing) was accounted for by including an adjustable constant parameter in the fit. The data analyses were conducted in Origin 7.0 (OriginLab, Northampton, MA, USA) with user-defined fitting functions.

### 2.9. Molecular Modelling and Simulation

#### 2.9.1. Modelling of the Isolated Proteins

The structure of ARM-PKP1 was derived from the crystallographic structure [49] present in the Protein Data Bank (PDB) and containing residues 244-700 (PDB entry: 1XM9). As in our previous works [52,53,54], we modeled two missing loops and did not include two small portions missing at both termini with respect to the protein construct used in our experiments (residues 237–704 of the intact protein). 

The structure of the RYBP model was retrieved from the AlphaFold database (UniProt identifier: Q8N488) [55,61], again as in our previous work [54]. The only ordered regions of this modeled protein are the zinc-finger domain (residues 23–47), the N-term helix (residue 59–69), and the β-hairpin motif (residue 165–175). Whereas the former and the latter are well-assessed binding spots of RYBP with some molecular partners [2], the N-term helix is a region with a high α-helical propensity predicted by AlphaFold to be fully folded and it has been recently shown that it is capable of interacting with a partner protein of RYBP, the RING1B-PCGF4 heterodimer [27]. All the other regions of the modeled RYBP are unfolded, and AlphaFold predicts that they have a low probability of adopting a structure (confidence score pLDDT < 60, where 0 is the minimum, and 100 is the maximum) [55].

#### 2.9.2. Docking Simulations

Docking simulations were performed by assuming that at least one of the known binding domains of the RYBP model should be involved in the association to ARM-PKP1. Three separate fragments of RYBP were considered: the fragments 20–55 (including the zinc-finger domain) and 50–85 (N-term helix), as well as the subsequent region possessing some propensity to form shorter helical turns and containing also the NLS of RYBP [28] and 145–180 (largely encompassing the C-terminal β-hairpin). All these fragments constitute rather independent domain units separated by more flexible regions. To minimize the bias in their size, they were chosen to have the same sequence length; as a consequence, fragments 20–55 and 50–85 overlapped for a small region.

Protein–protein docking simulations were performed by using three distinct algorithms, all with default options, as implemented in publicly available web servers: GalaxyDock [62], GRAMM [63], and HawkDock [64]. The latter was also used to perform more accurate, but slower, calculations based on molecular mechanics with the generalized Born surface area (MM/GBSA) methodology [65] to re-rank the best three docking poses found in the search performed by using either its own scoring function, HawkRank [66], or the other two algorithms. After re-ranking, only the most favorable pose was considered for each fragment for all the three prediction algorithms.

## 3. Results

### 3.1. PKP1 Interacted with RYBP In Cellulo

To test whether interaction between endogenous intact PKP1 and RYBP occurred within cells, we used cancer and non-cancer cell lines.

In patients with breast and lung cancers, the presence of high levels of PKP1 has been described [46], and such high expression has been correlated with a lower survival rate. Therefore, we used the MDA-MB-231 human breast adenocarcinoma cell line as a tumorigenic cell line.

On the other hand, carcinoma-associated fibroblasts (CAFs) are known to enhance tumor-cell invasion and migration through the initiation of EMT, and MRC-5 fibroblasts constitute one of the CAFs expressing alpha-smooth muscle actin. MRC-5 was established from the normal lung tissue of a 14-week old male fetus, i.e., at a stage when the lung is rich in fibroblasts. In addition, RYBP inhibits the progression and metastasis of lung cancer [19]. Moreover, the fact that the expression of PKP1 is high in fibroblasts and that PKP1 has been observed to be expressed in high amounts in lung cancers made the MRC-5 cell line a suitable candidate to check for the interaction between RYBP and PKP1 [46].

First, we performed IF experiments to address whether both proteins were expressed and colocalized within the same cellular compartments for the different cell lines (Figures S1 and S2). To this end, we used confocal microscopy that allowed us to see whether the two proteins were in the same place, and if so, it would be indicative of colocalization. We found that the two proteins were differently expressed in the two cell lines. In MRC-5, PKP1 and RYBP were found in the nucleus, as shown by the colocalization with DAPI, suggesting that they could interact within this cellular compartment. In the breast adenocarcinoma, the expression of both proteins was found mostly in the cytosol, suggesting that contrary to MRC-5, the interaction of PKP1 and RYBP may occur in the cytosol.

**Figure 2:**
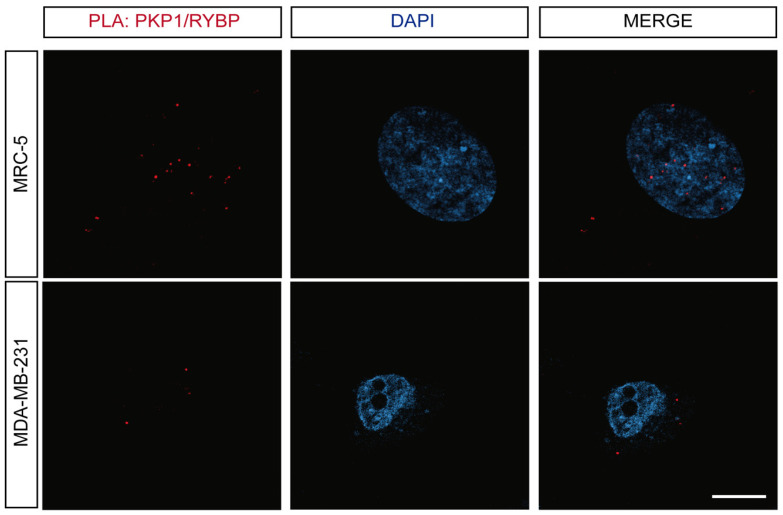
PKP1 interacted with RYBP in cellulo. PLAs of PKP1 with RYBP reveal the interactions between the two proteins in different cell lines. A representative experiment is shown (*n* = 5). Scale bar = 10 μm.

In order to confirm their in cellulo interaction, we used a technique known as proximity ligation assay (PLA), also referred to as the Duolink PLA technology. It is considered that PLA resolves the binding of endogenous proteins as it can detect whether the two proteins are separated by less than 40 nm of distance [67]. This technology has been broadly used to visualize the intracellular binding of proteins in a precise manner by several groups including ours, and it is broadly accepted in the field [33,53,68,69,70,71]. Proteins that interact directly will appear as red fluorescent spots, corresponding to the PLA signals. As suggested by the IF assays, PKP1and RYBP were colocalized at a short distance mostly within the nucleus, in the case of MRC-5, whereas for MDA-MB-231 cells, the interaction was restricted to the cytosol (Figure 2 and Figure S3). Moreover, the number of interactions per cell in the case of MDA-MB-231 cells was lower as compared with MRC-5. Therefore, the ability to interact of both proteins, and the location of such interaction, was cell-dependent. However, we cannot rule out unambiguously that the observed differences were not only due to the fact that cell lines were cancerous, but rather, could have also been due to some cancer tissue specificity.

To sum up, the confocal analysis of the IF experiments in cellulo indicated that PKP1 and RYBP were co-expressed in cells of different origin. Moreover, the direct interaction of the two proteins in different cell compartments was shown via PLA, and we could observe that it varied depending on the cell line analyzed.

### 3.2. Measuring the Affinity of ARM-PKP1 and RYBP In Vitro

As we had previously ascertained that there was binding between the two proteins in cellulo, next, we wanted to measure, quantitatively, such interaction in vitro by considering the armadillo region of PKP1, ARM-PKP1, as armadillo domains are distinctively involved in protein–protein interactions (PPIs). To that goal, we followed a three-part experimental approach. First, we used steady-state fluorescence and CD as spectroscopic techniques capable of detecting a possible binding and concomitant conformational changes in any of the macromolecules; second, we used fluorescence titrations to confirm, quantitatively, the association constant driving such PPIs; and finally, we used ITC to quantitatively measure the thermodynamic parameters of the binding.

**Figure 3:**
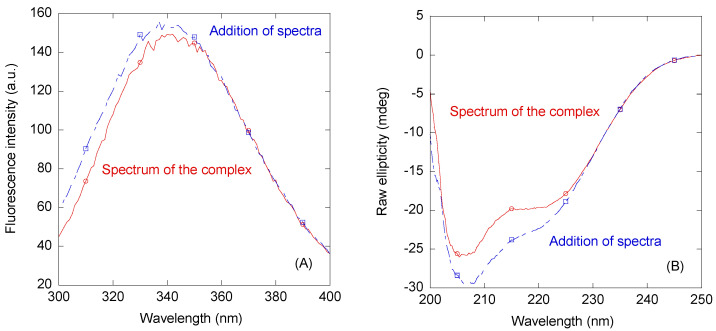
Binding of ARM-PKP1 to RYBP as monitored through spectroscopic techniques. (**A**) Fluorescence spectrum obtained through excitation at 280 nm of the RYBP/ARM-PKP1 complex and addition spectrum obtained from the sum of the spectra of the two isolated macromolecules. (**B**) Far-UV CD spectrum of the RYBP/ARM-PKP1 complex and addition spectrum obtained from the sum of the spectra of the two isolated macromolecules. All experiments were performed at 25 °C.

We first used steady-state fluorescence to determine whether there was a change in (i) the value of the maximum wavelength in the emission spectrum; (ii) the fluorescence intensity observed at that maximum wavelength; or (iii) both physical parameters when the spectrum of the complex was compared to that obtained from the addition of those of the two isolated proteins. A variation in fluorescence intensity via excitation at 280 nm was observed when the complex of RYBP with ARM-PKP1 was formed (Figure 3A), but there were no changes in the maximum wavelength of the spectrum of the complex. Similar variations were observed by excitation at 295 nm. These findings indicate that the tertiary structure around some of the aromatic residues of at least one of the proteins changed upon complex formation.

Next, we carried out far-UV CD measurements with the aim of further supporting the results obtained through fluorescence but, in this case, by following possible changes in the secondary structure of at least one of the proteins upon complex formation. The far-UV addition spectrum, obtained from the sum of the spectra of both polypeptide chains, was very different from that of the complex (Figure 3B). The differences could be attributed either to a relatively large number of aromatic residues involved in the binding or even to changes in the conformational propensities of RYBP (which is an IDP) and/or, although less likely, in those of ARM-PKP1 (which is folded) when the two proteins were bound.

**Figure 4:**
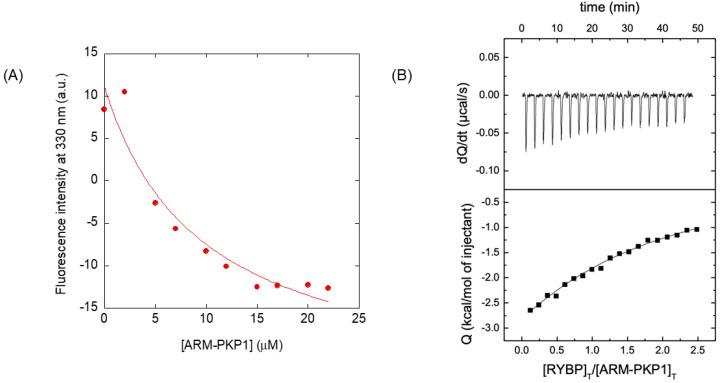
Binding of ARM-PKP1 to RYBP as monitored through biophysical techniques. (**A**) Titration curve monitoring the changes in the fluorescence at 330 nm when ARM-PKP1 was added to RYBP. The fluorescence intensity is the relative signal after removal of the corresponding blank. The line through the data is the fitting to Equation (1). (**B**) Calorimetric titration for the RYBP binding to ARM-PKP1. The upper panel shows the thermogram (thermal power as a function of time) and the lower panel shows the binding isotherm (ligand-normalized heat effects per injection as a function of the molar ratio in the calorimetric cell). The continuous line corresponds to the fitting curve according to an interaction model with a single ligand binding site. All replicates were carried out at 25 °C.

Finally, we carried out fluorescence titrations to quantitatively measure the binding affinity of the two proteins by keeping constant the concentration of RYBP and increasing the concentration of ARM-PKP1. The results (Figure 4A) indicate that the dissociation constant, *K*_d_, was 9 ± 5 µM. We also used ITC to determine the enthalpy and the entropy of the binding reaction (Figure 4B). The interaction was markedly exothermic (that is, it had a favorable enthalpic contribution and unfavorable entropic contribution to the Gibbs energy of binding), with Δ*H* = −11.9 ± 0.4 kcal mol^−1^, and the value of the *K*_d_ was 12 ± 2 µM. This value, although slightly greater than the one formerly obtained through fluorescence titration, was within the same order of magnitude, and the small difference could be easily attributed to the differences between the two experimental techniques. The stoichiometry of the reaction was 1:1, further supporting a direct, specific interaction between the two macromolecules.

To sum up, we concluded that there was evidence that RYBP could bind to ARM-PKP1 with a low micromolar affinity. However, we could not rule out that a more complex interaction may be taking place in cellulo, where other molecular partners could also be involved or affect their binding.

### 3.3. Structural Prediction of the Complex RYBP/ARM-PKP1

The prediction of the binding complex between RYBP and ARM-PKP1 is difficult due to the intrinsically disordered structure of RYBP, which is a mostly unstructured IDP. In contrast, ARM-PKP1 consists of a single folded domain with a highly organized structural architecture. Therefore, we sought to gain structural insight on their molecular complex by performing protein–protein docking calculations that considered the whole structure of ARM-PKP1 as the host and assuming that at least one of the known binding domains of the RYBP model should anchor to it. The simulations were carried out separately for three regions of RYBP: the fragments 20–55 (zinc-finger domain), 50–85 (N-term helix and NLS), and 145–180 (C-terminal β-hairpin). Three docking algorithms were used for the prediction: GalaxyDock [62], GRAMM [63], and HawkDock [64]. The results of these algorithms were compared by performing accurate MM/GBSA calculations to re-rank the best three docking poses found by each of them for every fragment and considering only the most favorable one as the final prediction.

The results obtained in our docking calculations are summarized in Table 1 and Figure S4. Among the docking programs used, there was an almost-consensus that all the fragments of RYBP considered had a favorable binding location in correspondence to the innermost region of the sagittal plane of ARM-PKP1. This location corresponded to the basic (positively charged) patch on the surface of ARM-PKP1 (Figure S5), which was already found to be the binding location for several molecular partners of this protein in our in silico results [52,53]. As the sole exception, HawkDock was the only predictor suggesting the possibility that the N-term α-helix and the β-hairpin motif would rather bind to other regions on the surface of ARM-PKP1, although with a lower docking score. Furthermore, in contrast with the other two predictors, it docked the zinc-finger domain in the uppermost region of the basic patch of ARM-PKP1. However, the docking poses found with HawkDock had a systematically less favorable MM/GBSA binding energy compared to the other two predictors. This suggests that the results provided by HawkDock reflect inaccuracies in the docking score for this specific biological system, and they are likely not relevant, being at variance with those of the other predictors. In this respect, the algorithm GalaxyDock appeared to be most accurate in finding docking poses with the best affinity. In line with the typical overestimation of the binding free energy provided by the MM/GBSA methodology [72], the binding scores calculated in simulation (Table 1) were consistent with the weak association observed in our experiments.

Whereas the basic patch clearly seems to be the most likely binding region for ARM-PKP1, our docking calculations were not decisive in determining which one is the corresponding binding region of RYBP. In fact, although the general trend indicated a higher probability for the zinc-finger domain and a lower probability for the N-term α-helix, the binding energies of the three fragments considered were too close to each other to draw a clear conclusion. Although there is not enough evidence to assess which is the single region of RYBP that ends up anchoring to the well-identified binding spot of ARM-PKP1, we cannot exclude that the simulation results reflect a real variety of possibilities as they may describe transient structures that would greatly reduce the large conformational funnel due to the high flexibility of the RYBP model within the energy landscape that dictates the formation of the final RYBP/ARM-PKP1 complex. Unfortunately, even more accurate simulation techniques (e.g., molecular dynamics simulations) could be of little help in this case due to the relatively large size and conformational flexibility of the RYBP model. More importantly, any other simulation technique would still strongly depend on the starting conformation of the RYBP/ARM-PKP1 complex provided by molecular docking or any other sort of preliminary modeling methodology.

Besides providing a rank for the docking poses found by docking algorithms that use different scoring functions, another advantage in the use of the MM/GBSA methodology is that it gives the possibility to decompose into per-residue contribution the calculated binding energy [72]. Interestingly, this allowed us to predict some important residues of ARM-PKP1 that are crucial for the binding of RYBP as suggested by our in silico findings, irrespective of the persisting uncertainty about the most favorable regions of the latter that may anchor to such hot-spots. In fact, the key residue Trp355 (Figure S5), and possibly a few other solvent-exposed aromatic residues (e.g., His459, Tyr463) of ARM-PKP1, are common to the binding poses found in each of the three docking fragments of the RYBP model considered and, therefore, are expected to play an important role in the association between the two proteins.

## 4. Discussion

We have recently shown that PADI4 binds to ARM-PKP1 [54] and RYBP [33]. Furthermore, both proteins are implicated in the progression of several cancer types [18,38,46,73,74], and they can act as oncogenes or tumor suppressors. In fact, several studies have shown that the expression of RYBP is dysregulated in various human tumor tissues, including prostate [75,76], lung [77], liver, breast, hepatocellular carcinoma, glioblastoma [78], Hodgkin lymphoma, and cervical cancer [17,19,79,80,81,82,83]. In general terms, RYBP expression appears to be downregulated in those cancer types, and low RYBP expression is associated with poor prognosis [77,82,84,85]. On the other hand, PKP1 appears implicated in the development of prostate, lung, and breast cancers, among others [35,38,39,46,73,86]. Based on these data, we hypothesized that PKP1 and RYBP could interact directly with each other, at least in some of those cancer cell lines. To test the hypothesis of a well-defined complex between the two proteins, we carried out several experiments on breast cancer cells as opposed to lung-developing healthy cell lines. Interestingly, RYBP binds to, and upregulates, fibronectin type III and ankyrin-repeat-domain-1 protein in tumor cells to induce apoptosis via the JNK-AP1 signaling pathway [13]. As the ankyrin domain is a short helical motif consisting in tandem repeats and involved in PPIs, it is not entirely surprising that RYBP could interact as well with PKP1.

PKP1 has an ARM domain, and since these domains are known to intervene in PPIs, we studied, in vitro and in silico, the binding properties of ARM-PKP1 and RYBP. The binding interface of ARM-PKP1 is the basic patch on its molecular surface, as found in our docking simulations. This polypeptide patch was proposed to be the main binding region of PKP1 with other molecular partners [52,53]. In fact, NUPR1, another IDP, was also found to bind to this same region through a highly acidic region at its N terminus [53], and Trp355 of ARM-PKP1 was also involved in such binding to NUPR1 as it was found in the interaction with the RYBP model. This result provides a rationale for the fluorescence results as other aromatic residues of ARM-PKP1 also participate in the binding. Furthermore, it would also contribute to explain the fact that the stoichiometry of the binding reaction was 1:1, which is an observation that could not be otherwise supported by a less specific anchoring to the ARM-repeat helical structures of PKP1. In contrast, the contribution to the binding of the PPIs provided by the most disordered regions of RYBP, which were not considered in our simulations, remains unclear. In principle, these regions might either favor or oppose to the binding to ARM-PKP1. However, we note that the two longest disordered regions of the structure of the RYBP model could not hinder the anchoring to the basic patch of ARM-PKP1. In fact, the unfolded region of the RYBP model rich in Lys and Arg residues has an electrostatic repulsion with the positively charged patch of ARM-PKP1. Similarly, the C-terminal region of RYBP rich in Ser and Thr residues and the basic patch of ARM-PKP1 will have an unfavorable binding affinity for each other because of the considerable energetic cost of their desolvation as the former is highly polar and the latter is charged.

The measured *K*_d_ for the RYBP/ARM-PKP1 complex (~10 µM) is comparable to that observed for ARM-PKP1 in complexes with other proteins, including NUPR1 (~10 µM) [53], or well-folded proteins such as the SAM of p73 (~5 µM) [52] and PADI4 (1.4 µM) [54]. On the other hand, the value of *K*_d_ is also similar to that measured in the interaction between RYBP and PADI4 (~10 µM) [33]. The strengths of the interactions with the different molecular partners of any of these two proteins were similar in all cases, indicating that despite a moderately favorable binding, the affinity might be enough to drive (or at least contribute to impact) a proper regulation in several pathways where ARM-PKP1 or RYBP intervene, determining a high specificity. We also note that an overall negative entropy was observed in the binding between ARM-PKP1 and PKP1; as hydrophobic and electrostatic interactions are generally accompanied by positive entropy changes, this implies that other contributions (such as conformational changes in RYBP, which could help to explain the far-UV CD data in Figure 3B) should be associated with the large negative entropy detected in our experiments [87]. At this stage, it is important to indicate that the relevance and specificity of a PPI depends not only on the binding affinity but also on the concentration of the interacting proteins. Thus, low-affinity interactions might be relevant if the interacting proteins were present at significant concentrations while high-affinity interactions might be irrelevant if any of the interacting proteins was poorly expressed. However, at this stage, we cannot rule out that the physiological interaction between the two proteins within a cell could involve another scaffolding biomolecule, forming a ternary complex. Still, we have provided pieces of evidence supporting the direct interaction between RYBP and ARM-PKP1 in our in vitro results.

PKP1 is associated with mRNA ribonucleoprotein particles and ribosomal proteins [35,88,89], and its altered expression is a frequent and critical event in the progression of several cancers [35,36,90,91], indicating that it has key roles in cell proliferation, differentiation, and migration. It is interesting to note that to date, no NLS has yet been identified within the sequence of PKP1 [92]. Although it is thought that its amino-terminal region might possibly be involved in its nuclear localization, it has not been ruled out that the nuclear translocation of PKP1 could rely on a different mechanism and might involve its binding as a cargo to another protein that is being translocated [92]. Since RYBP binds to some of the proteins involved in the nuclear translocation machinery [28], it is tempting to suggest that the complex RYBP/ARM-PKP1 could be transported within the nucleus as our docking simulations exclude that the NLS of RYBP is involved in the binding to ARM-PKP1.

On the other hand, PKP1 also forms desmosomes, protein complexes crucial to maintain cell–cell adhesion and the integrity of tissues, together with transmembrane cadherins and desmoplakin [93]. Desmosomes have key roles in cell growth, adhesion, and invasiveness (upon migration) and in apoptosis [42,94]. Our IF and PLA findings indicate that the interaction between the two proteins can occur either in the nucleus or in the cytoplasm (Figure 2 and Figure S1). It has been shown that RYBP inhibits the progression and metastasis of lung cancer by suppressing EGFR signaling and EMT [19], and high levels of PKP1 are important to maintain a high expression of vimentin, which is a key regulator of EMT and metastasis [46]. Thus, on the basis of our results describing the binding between the two proteins, we could also hypothesize that the interaction between RYBP and PKP1 could act as a rescuer of the excess of PKP1, decreasing the amount of EMT in tumor cells.

## 5. Conclusions

In this work, we have unambiguously demonstrated a direct association between RYBP and PKP1, two proteins separately known to be involved in several types of tumor processes. The interaction of these two proteins is complex as it could be expected due to the highly disordered nature of RYBP. Furthermore, it takes place involving the structurally organized ARM-repeat domain of PKP1. In the context of the current knowledge of the biological pathways both proteins participate in, we propose a possible novel role for RYBP as a rescuer in some tumor cells with a high content of PKP1. On the other hand, our results suggest new possible functions for the non-junctional forms of PKP1, expanding the variety of its interaction partners and possibly its mission in the diverse pathways it is involved in. Our findings also open the venue for identifying new biological mechanisms based on the RYBP/ARM-PKP1 interaction. Those mechanisms may become the target for the development of new pharmacological drugs to impact cancer development and progression among other therapeutic applications.

## Figures and Tables

**Table 1:** Binding energy calculated by using the MM/GBSA methodology for the most favorable docking poses of mostly-folded fragments of the RYBP model on the surface of ARM-PKP1.

	Total and Per-Residue ^(a)^ Binding Energy (kcal mol^−1^)		
Docking Algorithm	Fragment 20–55 (Zinc-Finger Domain)	Fragment 50–85 (N-Terminal α-Helix and NLS)	Fragment 145–180 (C-Terminal β-Hairpin)
HawkDock	Total, −28.40 Arg83, −4.18 His216, −3.38 Tyr220, −2.67 Pro434, −2.01 Gln309, −1.84	Total, −21.47 Gln62, −2.81 Pro157, −2.7 Val154, −2.16 Glu153, −1.53 Phe140, −1.42	Total, −6.27 Thr179, −4.17 Ile125, −2.54 Asn182, −1.79 Pro227, −1.78 Glu225, −1.64
GRAMM	Total, −36.06 Trp355, −4.32 Asn460, −3.86 His459, −3.01 Asp589, −2.6 Tyr463, −2.44	Total, −22.58 Tyr463, −4.56 Phe404, −4.28 Glu452, −2.77 Trp355, −2.2 His459, −2.1	Total, −11.53 Arg411, −3.05 Trp355, −2.25 Asn600, −2.24 Asn629, −1.96 Tyr463, −1.86
GalaxyDock	Total, −40.73 Trp355, −4.79 Asp541, −3.68 Arg317, −3.53 Glu277, −3.15 Asp589, −2.83	Total, −25.93 Trp355, −4.59 Phe316, −3.81 Asn412, −2.79 Phe274, −2.64 Tyr463, −2.41	Total, −39.88 Glu545, −5.17 Trp355, −3.91 Asn356, −2.83 Ser359, −2.26 Glu401, −1.95

^(a)^ Five most important binding residues.

## Data Availability

The data and materials are available from the authors upon reasonable request.

## Supplementary Materials

The following supporting information can be downloaded at: https://www.mdpi.com/article/10.3390/biom14050561/s1, Figure S1: IF images of RYBP and PKP1. RYBP (red), PKP1 (green) and DAPI (blue) in the two used cell lines. Scale bar: 10 µm. Figure S2: Magnification of RYBP and PKP1 IF images. RYBP (red), PKP1 (green), and DAPI (blue) in the two used cell lines. Scale bar: 10 µm. Figure S3: PLAs of RYBP and PKP1. PLA was performed in MRC-5 and MDA-MB-231 cells. A representative experiment is shown (*n* = 5). Scale bar: 10 µm. Figure S4: In silico experiments of the complex between RYBP and ARM-PKP1. (**A**) Binding locations of the fragments of RYBP on the surface ARM-PKP1 found in molecular docking simulations. Columns from left to right: fragment 20–55 (zinc-finger domain), 50–85 (N-term helix and NLS), and 145–180 (C-terminal β-hairpin). Rows from top to bottom: predictions of HawkDock (cyan), GRAMM (purple), GalaxyDock (orange). (**B**) Location of the basic binding patch on the inner surface of ARM-PKP1, and aromatic residues involved in the binding. In all cases ARM-PKP1 is shown with the innermost regions and the basic patch in front (see also Figure S5). Figure S5: Basic patch on the surface of ARM-PKP1 and key aromatic residues involved in the binding. The protein is shown (left) with the innermost regions and the basic patch in front, and (right) rotated by 90°. The figure was colored by residue type: hydrophobic residues in black; basic residues in red; and acidic residues in blue.

## References

[1] Garcia E., Marcos-Gutiérrez C., Del Mar Lorente M., Moreno J.C., Vidal M. (1999). RYBP, a New Repressor Protein That Interacts with Components of the Mammalian Polycomb Complex, and with the Transcription Factor YY1. EMBO J..

[2] Simoes da Silva C.J., Simón R., Busturia A. (2018). Epigenetic and Non-Epigenetic Functions of the RYBP Protein in Development and Disease. Mech. Ageing Dev..

[3] Bajusz I., Henry S., Sutus E., Kovács G., Pirity M.K. (2019). Evolving Role of RING1 and YY1 Binding Protein in the Regulation of Germ-Cell-Specific Transcription. Genes.

[4] Gao Z., Zhang J., Bonasio R., Strino F., Sawai A., Parisi F., Kluger Y., Reinberg D. (2012). PCGF Homologs, CBX Proteins, and RYBP Define Functionally Distinct PRC1 Family Complexes. Mol. Cell.

[5] de Napoles M., Mermoud J.E., Wakao R., Tang Y.A., Endoh M., Appanah R., Nesterova T.B., Silva J., Otte A.P., Vidal M. (2004). Polycomb Group Proteins Ring1A/B Link Ubiquitylation of Histone H2A to Heritable Gene Silencing and X Inactivation. Dev. Cell.

[6] Simon J.A., Kingston R.E. (2009). Mechanisms of Polycomb Gene Silencing: Knowns and Unknowns. Nat. Rev. Mol. Cell Biol..

[7] Bejarano F., González I., Vidal M., Busturia A. (2005). The Drosophila RYBP Gene Functions as a Polycomb-Dependent Transcriptional Repressor. Mech. Dev..

[8] Tavares L., Dimitrova E., Oxley D., Webster J., Poot R., Demmers J., Bezstarosti K., Taylor S., Ura H., Koide H. (2012). RYBP-PRC1 Complexes Mediate H2A Ubiquitylation at Polycomb Target Sites Independently of PRC2 and H3K27me3. Cell.

[9] Blackledge N.P., Klose R.J. (2021). The Molecular Principles of Gene Regulation by Polycomb Repressive Complexes. Nat. Rev. Mol. Cell Biol..

[10] Schlisio S., Halperin T., Vidal M., Nevins J.R. (2002). Interaction of YY1 with E2Fs, Mediated by RYBP, Provides a Mechanism for Specificity of E2F Function. EMBO J..

[11] Fursova N.A., Blackledge N.P., Nakayama M., Ito S., Koseki Y., Farcas A.M., King H.W., Koseki H., Klose R.J. (2019). Synergy between Variant PRC1 Complexes Defines Polycomb-Mediated Gene Repression. Mol. Cell.

[12] Rose N.R., King H.W., Blackledge N.P., Fursova N.A., Ember K.J., Fischer R., Kessler B.M., Klose R.J. (2016). RYBP Stimulates PRC1 to Shape Chromatin-Based Communication between Polycomb Repressive Complexes. Elife.

[13] Ma W., Zhang X., Li M., Ma X., Huang B., Chen H., Chen D. (2016). Proapoptotic RYBP Interacts with FANK1 and Induces Tumor Cell Apoptosis through the AP-1 Signaling Pathway. Cell. Signal..

[14] Zheng L., Schickling O., Peter M.E., Lenardo M.J. (2001). The Death Effector Domain-Associated Factor Plays Distinct Regulatory Roles in the Nucleus and Cytoplasm. J. Biol. Chem..

[15] Danen-van Oorschot A.A.A.M., Voskamp P., Seelen M.C.M.J., van Miltenburg M.H.A.M., Bolk M.W., Tait S.W., Boesen-de Cock J.G.R., Rohn J.L., Borst J., Noteborn M.H.M. (2004). Human Death Effector Domain-Associated Factor Interacts with the Viral Apoptosis Agonist Apoptin and Exerts Tumor-Preferential Cell Killing. Cell Death Differ..

[16] Gervais F.G., Singaraja R., Xanthoudakis S., Gutekunst C.A., Leavitt B.R., Metzler M., Hackam A.S., Tam J., Vaillancourt J.P., Houtzager V. (2002). Recruitment and Activation of Caspase-8 by the Huntingtin-Interacting Protein Hip-1 and a Novel Partner Hippi. Nat. Cell Biol..

[17] Chen D., Zhang J., Li M., Rayburn E.R., Wang H., Zhang R. (2009). RYBP Stabilizes P53 by Modulating MDM2. EMBO Rep..

[18] Morinaka T., Sakai N., Takayashiki T., Kuboki S., Takano S., Ohira G., Matsubara H., Ohtsuka M. RYBP Contributes to Improved Prognosis in Colorectal Cancer via Regulation of Cell Cycle, Apoptosis and Oxaliplatin Sensitivity. https://www.spandidos-publications.com/10.3892/ijo.2023.5568.

[19] Dinglin X., Ding L., Li Q., Liu Y., Zhang J., Yao H. (2017). RYBP Inhibits Progression and Metastasis of Lung Cancer by Suppressing EGFR Signaling and Epithelial-Mesenchymal Transition. Transl. Oncol..

[20] Their J.P. (2002). Epithelial–Mesenchymal Transitions in Tumour Progression. Nat. Rev. Cancer.

[21] Yang J., Weinberg R.A. (2008). Epithelial-Mesenchymal Transition: At the Crossroads of Development and Tumor Metastasis. Dev. Cell.

[22] Arrigoni R., Alam S.L., Wamstad J.A., Bardwell V.J., Sundquist W.I., Schreiber-Agus N. (2006). The Polycomb-Associated Protein Rybp Is a Ubiquitin Binding Protein. FEBS Lett..

[23] Zhao J., Wang M., Chang L., Yu J., Song A., Liu C., Huang W., Zhang T., Wu X., Shen X. (2020). RYBP/YAF2-PRC1 Complexes and Histone H1-Dependent Chromatin Compaction Mediate Propagation of H2AK119ub1 during Cell Division. Nat. Cell Biol..

[24] Fereres S., Simón R., Mohd-Sarip A., Verrijzer C.P., Busturia A. (2014). DRYBP Counteracts Chromatin-Dependent Activation and Repression of Transcription. PLoS ONE.

[25] Neira J.L., Román-Trufero M., Contreras L.M., Prieto J., Singh G., Barrera F.N., Renart M.L., Vidal M. (2009). The Transcriptional Repressor RYBP Is a Natively Unfolded Protein Which Folds upon Binding to DNA. Biochemistry.

[26] Alam S.L., Sun J., Payne M., Welch B.D., Blake B.K., Davis D.R., Meyer H.H., Emr S.D., Sundquist W.I. (2004). Ubiquitin Interactions of NZF Zinc Fingers. EMBO J..

[27] Silva C.S., Mariño Pérez L., García Ferrer I., Dieryck I., Pessey O., Boeri Erba E., Ringkjøbing Jensen M., Macia M. (2023). A Previously-Unrecognized Motif of Transcription Factor RYBP, Hotspot of Cancer-Related Mutations, Is Essential for the Integrity of Polycomb Repressive Complex 1. bioRxiv.

[28] Neira J.L., Jiménez-Alesanco A., Rizzuti B., Velazquez-Campoy A. (2021). The Nuclear Localization Sequence of the Epigenetic Factor RYBP Binds to Human Importin alpha3. Biochim. Biophys. Acta Proteins Proteom..

[29] Gsponer J., Futschik M.E., Teichmann S.A., Babu M.M. (2008). Tight Regulation of Unstructured Proteins: From Transcript Synthesis to Protein Degradation. Science.

[30] Berlow R.B., Dyson H.J., Wright P.E. (2018). Expanding the Paradigm: Intrinsically Disordered Proteins and Allosteric Regulation. J. Mol. Biol..

[31] Xie H., Vucetic S., Iakoucheva L.M., Oldfield C.J., Dunker A.K., Uversky V.N., Obradovic Z. (2007). Functional Anthology of Intrinsic Disorder. 1. Biological Processes and Functions of Proteins with Long Disordered Regions. J. Proteome Res..

[32] Babu M.M., van der Lee R., de Groot N.S., Gsponer J. (2011). Intrinsically Disordered Proteins: Regulation and Disease. Curr. Opin. Struct. Biol..

[33] Araujo-Abad S., Fuentes-Baile M., Rizzuti B., Bazán J.F., Villamarin-Ortiz A., Saceda M., Fernández E., Vidal M., Abian O., Velazquez-Campoy A. (2023). The Intrinsically Disordered, Epigenetic Factor RYBP Binds to the Citrullinating Enzyme PADI4 in Cancer Cells. Int. J. Biol. Macromol..

[34] Bonné S., Van Hengel J., Nollet F., Kools P., Van Roy F. (1999). Plakophilin-3, a Novel Armadillo-like Protein Present in Nuclei and Desmosomes of Epithelial Cells. J. Cell Sci..

[35] Fischer-Kešo R., Breuninger S., Hofmann S., Henn M., Röhrig T., Ströbel P., Stoecklin G., Hofmann I. (2014). Plakophilins 1 and 3 Bind to FXR1 and Thereby Influence the MRNA Stability of Desmosomal Proteins. Mol. Cell. Biol..

[36] Hofmann I., Casella M., Schnölzer M., Schlechter T., Spring H., Franke W.W. (2006). Identification of the Junctional Plaque Protein Plakophilin 3 in Cytoplasmic Particles Containing RNA-Binding Proteins and the Recruitment of Plakophilins 1 and 3 to Stress Granules. Mol. Biol. Cell.

[37] Bonné S., Gilbert B., Hatzfeld M., Chen X., Green K.J., Van Roy F. (2003). Defining Desmosomal Plakophilin-3 Interactions. J. Cell Biol..

[38] Martin-Padron J., Boyero L., Rodriguez M.I., Andrades A., Díaz-Cano I., Peinado P., Baliñas-Gavira C., Alvarez-Perez J.C., Coira I.F., Fárez-Vidal M.E. (2019). Plakophilin 1 Enhances MYC Translation, Promoting Squamous Cell Lung Cancer. Oncogene.

[39] Boyero L., Martin-Padron J., Fárez-Vidal M.E., Rodriguez M.I., Andrades Á., Peinado P., Arenas A.M., Ritoré-Salazar F., Alvarez-Perez J.C., Cuadros M. (2022). PKP1 and MYC Create a Feedforward Loop Linking Transcription and Translation in Squamous Cell Lung Cancer. Cell. Oncol..

[40] Bass-Zubek A.E., Godsel L.M., Delmar M., Green K.J. (2009). Plakophilins: Multifunctional Scaffolds for Adhesion and Signaling. Curr. Opin. Cell Biol..

[41] Kapprell H.P., Owaribe K., Franke W.W. (1988). Identification of a Basic Protein of Mr 75,000 as an Accessory Desmosomal Plaque Protein in Stratified and Complex Epithelia. J. Cell Biol..

[42] Hatzfeld M. (2007). Plakophilins: Multifunctional Proteins or Just Regulators of Desmosomal Adhesion?. Biochim. Biophys. Acta.

[43] Sanchez-Palencia A., Gomez-Morales M., Gomez-Capilla J.A., Pedraza V., Boyero L., Rosell R., Fárez-Vidal M.E. (2011). Gene Expression Profiling Reveals Novel Biomarkers in Nonsmall Cell Lung Cancer. Int. J. Cancer.

[44] Gómez-Morales M., Cámara-Pulido M., Miranda-León M.T., Sánchez-Palencia A., Boyero L., Gómez-Capilla J.A., Fárez-Vidal M.E. (2013). Differential Immunohistochemical Localization of Desmosomal Plaque-Related Proteins in Non-Small-Cell Lung Cancer. Histopathology.

[45] Galindo I., Gómez-Morales M., Díaz-Cano I., Andrades Á., Caba-Molina M., Teresa Miranda-León M., Pablo Medina P., Martín-Padron J., Esther Fárez-Vidal M., Gomez-Morales M.G. (2020). The Value of Desmosomal Plaque-Related Markers to Distinguish Squamous Cell Carcinoma and Adenocarcinoma of the Lung. Upsala J. Med. Sci..

[46] Li K., Wu R., Zhou M., Tong H., Luo K.Q. (2021). Desmosomal Proteins of DSC2 and PKP1 Promote Cancer Cells Survival and Metastasis by Increasing Cluster Formation in Circulatory System. Sci. Adv..

[47] Haase D., Cui T., Yang L., Ma Y., Liu H., Theis B., Petersen I., Chen Y. (2019). Plakophilin 1 Is Methylated and Has a Tumor Suppressive Activity in Human Lung Cancer. Exp. Mol. Pathol..

[48] Peifer M., Berg S., Reynolds A.B. (1994). A Repeating Amino Acid Motif Shared by Proteins with Diverse Cellular Roles. Cell.

[49] Choi H.J., Weis W.I. (2005). Structure of the Armadillo Repeat Domain of Plakophilin 1. J. Mol. Biol..

[50] Jernigan K.K., Bordenstein S.R. (2015). Tandem-Repeat Protein Domains across the Tree of Life. PeerJ.

[51] Giudici A.M., Hernández-Cifre J.G., Cámara-Artigas A., Hornos F., Martínez-Rodríguez S., Carlos Alvarez-Pérez J., Díaz-Cano I., Esther Fárez-Vidal M., Neira J.L. (2020). The Isolated Armadillo-Repeat Domain of Plakophilin 1 Is a Monomer in Solution with a Low Conformational Stability. J. Struct. Biol..

[52] Neira J.L., Rizzuti B., Ortega-Alarcón D., Giudici A.M., Abián O., Fárez-Vidal M.E., Velázquez-Campoy A. (2021). The Armadillo-Repeat Domain of Plakophilin 1 Binds the C-Terminal Sterile Alpha Motif (SAM) of P73. Biochim. Biophys. Acta Gen. Subj..

[53] Santofimia-Castaño P., Rizzuti B., Pey A.L., Fárez-Vidal M.E., Iovanna J.L., Neira J.L. (2021). Intrinsically Disordered Protein NUPR1 Binds to the Armadillo-Repeat Domain of Plakophilin 1. Int. J. Biol. Macromol..

[54] Neira J.L., Rizzuti B., Araujo-Abad S., Abian O., Fárez-Vidal M.E., Velazquez-Campoy A., de Juan Romero C. (2023). The Armadillo-Repeat Domain of Plakophilin 1 Binds to Human Enzyme PADI4. Biochim. Biophys. Acta Proteins Proteom..

[55] Jumper J., Evans R., Pritzel A., Green T., Figurnov M., Ronneberger O., Tunyasuvunakool K., Bates R., Žídek A., Potapenko A. (2021). Highly Accurate Protein Structure Prediction with AlphaFold. Nature.

[56] Gill S.C., von Hippel P.H. (1989). Calculation of Protein Extinction Coefficients from Amino Acid Sequence Data. Anal. Biochem..

[57] Neira J.L., Hornos F., Bacarizo J., Cámara-Artigás A., Gómez J. (2016). The Monomeric Species of the Regulatory Domain of Tyrosine Hydroxylase Has a Low Conformational Stability. Biochemistry.

[58] Birdsall B., King R.W., Wheeler M.R., Lewis C.A., Goode S.R., Dunlap R.B., Roberts G.C.K. (1983). Correction for Light Absorption in Fluorescence Studies of Protein-Ligand Interactions. Anal. Biochem..

[59] Beckett D. (2011). Measurement and Analysis of Equilibrium Binding Titrations: A Beginner’s Guide. Methods Enzymol..

[60] Royer C.A., Scarlata S.F. (2008). Fluorescence Approaches to Quantifying Biomolecular Interactions. Methods Enzymol..

[61] Saldanõ T., Escobedo N., Marchetti J., Zea D.J., Mac Donagh J., Velez Rueda A.J., Gonik E., Garciá Melani A., Novomisky Nechcoff J., Salas M.N. (2022). Impact of Protein Conformational Diversity on AlphaFold Predictions. Bioinformatics.

[62] Shin W.H., Seok C. (2012). GalaxyDock: Protein-Ligand Docking with Flexible Protein Side-Chains. J. Chem. Inf. Model..

[63] Singh A., Copeland M.M., Kundrotas P.J., Vakser I.A. (2024). GRAMM Web Server for Protein Docking. Methods Mol. Biol..

[64] Weng G., Wang E., Wang Z., Liu H., Zhu F., Li D., Hou T. (2019). HawkDock: A Web Server to Predict and Analyze the Protein-Protein Complex Based on Computational Docking and MM/GBSA. Nucleic Acids Res..

[65] Chen F., Liu H., Sun H., Pan P., Li Y., Li D., Hou T. (2016). Assessing the Performance of the MM/PBSA and MM/GBSA Methods. 6. Capability to Predict Protein–Protein Binding Free Energies and Re-Rank Binding Poses Generated by Protein–Protein Docking. Phys. Chem. Chem. Phys..

[66] Feng T., Chen F., Kang Y., Sun H., Liu H., Li D., Zhu F., Hou T. (2017). HawkRank: A New Scoring Function for Protein-Protein Docking Based on Weighted Energy Terms. J. Cheminform..

[67] Alam M.S. (2022). Proximity Ligation Assay (PLA). Methods Mol. Biol..

[68] Araujo-Abad S., Neira J.L., Rizzuti B., García-Morales P., de Juan Romero C., Santofimia-Castaño P., Iovanna J. (2023). Intrinsically Disordered Chromatin Protein NUPR1 Binds to the Enzyme PADI4. J. Mol. Biol..

[69] Araujo-Abad S., Rizzuti B., Villamarin-Ortiz A., Pantoja-Uceda D., Moreno-Gonzalez C.M., Abian O., Velazquez-Campoy A., Neira J.L., de Juan Romero C. (2023). New Insights into Cancer: MDM2 Binds to the Citrullinating Enzyme PADI4. Protein Sci..

[70] Santofimia-Castaño P., Rizzuti B., Pey Á.L., Soubeyran P., Vidal M., Urrutia R., Iovanna J.L., Neira J.L. (2017). Intrinsically Disordered Chromatin Protein NUPR1 Binds to the C-Terminal Region of Polycomb RING1B. Proc. Natl. Acad. Sci. USA.

[71] Vuono E., Ramirez-Medina E., Silva E., Berggren K., Rai A., Espinoza N., Borca M.V., Gladue D.P. (2023). The Interaction between the DOCK7 Protein and the E2 Protein of Classical Swine Fever Virus Is Not Involved with Viral Replication or Pathogenicity. Viruses.

[72] Rizzuti B., Grande F. (2020). Virtual Screening in Drug Discovery: A Precious Tool for a Still-Demanding Challenge. Protein Homeostasis Diseases: Mechanisms and Novel Therapies.

[73] Kim M., Reidenbach S., Schlechter T., Rothmann A.C., Will R., Hofmann I. (2023). Plakophilin 1 Deficiency in Prostatic Tumours Is Correlated with Immune Cell Recruitment and Controls the Up-Regulation of Cytokine Expression Post-Transcriptionally. FEBS J..

[74] Demirag G.G., Sullu Y., Yucel I. (2012). Expression of Plakophilins (PKP1, PKP2, and PKP3) in Breast Cancers. Med. Oncol..

[75] Taylor B.S., Schultz N., Hieronymus H., Gopalan A., Xiao Y., Carver B.S., Arora V.K., Kaushik P., Cerami E., Reva B. (2010). Integrative Genomic Profiling of Human Prostate Cancer. Cancer Cell.

[76] Krohn A., Seidel A., Burkhardt L., Bachmann F., Mader M., Grupp K., Eichenauer T., Becker A., Adam M., Graefen M. (2013). Recurrent Deletion of 3p13 Targets Multiple Tumour Suppressor Genes and Defines a Distinct Subgroup of Aggressive ERG Fusion-Positive Prostate Cancers. J. Pathol..

[77] Voruganti S., Xu F., Qin J.J., Guo Y., Sarkar S., Gao M., Zheng Z., Wang M.H., Zhou J., Qian B. (2015). RYBP Predicts Survival of Patients with Non-Small Cell Lung Cancer and Regulates Tumor Cell Growth and the Response to Chemotherapy. Cancer Lett..

[78] Li G., Warden C., Zou Z., Neman J., Krueger J.S., Jain A., Jandial R., Chen M. (2013). Altered Expression of Polycomb Group Genes in Glioblastoma Multiforme. PLoS ONE.

[79] Lando M., Holden M., Bergersen L.C., Svendsrud D.H., Stokke T., Sundfør K., Glad I.K., Kristensen G.B., Lyng H. (2009). Gene Dosage, Expression, and Ontology Analysis Identifies Driver Genes in the Carcinogenesis and Chemoradioresistance of Cervical Cancer. PLoS Genet..

[80] Lando M., Wilting S.M., Snipstad K., Clancy T., Bierkens M., Aarnes E.K., Holden M., Stokke T., Sundfør K., Holm R. (2013). Identification of Eight Candidate Target Genes of the Recurrent 3p12-P14 Loss in Cervical Cancer by Integrative Genomic Profiling. J. Pathol..

[81] Sánchez-Beato M., Sánchez E., García J.F., Pérez-Rosado A., Montoya M.C., Fraga M., Artiga M.J., Navarrete M., Abraira V., Morente M. (2004). Abnormal PcG Protein Expression in Hodgkin’s Lymphoma. Relation with E2F6 and NFkappaB Transcription Factors. J. Pathol..

[82] Wang W., Cheng J., Qin J.J., Voruganti S., Nag S., Fan J., Gao Q., Zhang R. (2014). RYBP Expression Is Associated with Better Survival of Patients with Hepatocellular Carcinoma (HCC) and Responsiveness to Chemotherapy of HCC Cells in Vitro and in Vivo. Oncotarget.

[83] Zhan S., Wang T., Ge W., Li J. (2018). Multiple Roles of Ring 1 and YY1 Binding Protein in Physiology and Disease. J. Cell. Mol. Med..

[84] Zhou H., Li J., Zhang Z., Ye R., Shao N., Cheang T., Wang S. (2016). RING1 and YY1 Binding Protein Suppresses Breast Cancer Growth and Metastasis. Int. J. Oncol..

[85] Zhu X., Yan M., Luo W., Liu W., Ren Y., Bei C., Tang G., Chen R., Tan S. (2017). Expression and Clinical Significance of PcG-Associated Protein RYBP in Hepatocellular Carcinoma. Oncol. Lett..

[86] Cheung I.Y., Feng Y., Danis K., Shukla N., Meyers P., Ladanyi M., Cheung N.K.V. (2007). Novel Markers of Subclinical Disease for Ewing Family Tumors from Gene Expression Profiling. Clin. Cancer Res..

[87] Iwahara J., Esadze A., Zandarashvili L. (2015). Physicochemical Properties of Ion Pairs of Biological Macromolecules. Biomolecules.

[88] Sobolik-Delmaire T., Reddy R., Pashaj A., Roberts B.J., Wahl J.K. (2010). Plakophilin-1 Localizes to the Nucleus and Interacts with Single-Stranded DNA. J. Investig. Dermatol..

[89] Hatzfeld M., Haffner C., Schulze K., Vinzens U. (2000). The Function of Plakophilin 1 in Desmosome Assembly and Actin Filament Organization. J. Cell Biol..

[90] Sobolik-Delmaire T., Katafiasz D., Keim S.A., Mahoney M.G., Wahl J.K. (2007). Decreased Plakophilin-1 Expression Promotes Increased Motility in Head and Neck Squamous Cell Carcinoma Cells. Cell Commun. Adhes..

[91] Yang C., Fischer-Kešo R., Schlechter T., Ströbel P., Marx A., Hofmann I. (2015). Plakophilin 1-Deficient Cells Upregulate SPOCK1: Implications for Prostate Cancer Progression. Tumour Biol..

[92] Hatzfeld M. (2010). A Nuclear Function for Plakophilin-1 in the DNA Damage Response?. J. Investig. Dermatol..

[93] Franzen C.A., Todorović V., Desai B.V., Mirzoeva S., Yang X.J., Green K.J., Pelling J.C. (2012). The Desmosomal Armadillo Protein Plakoglobin Regulates Prostate Cancer Cell Adhesion and Motility through Vitronectin-Dependent Src Signaling. PLoS ONE.

[94] Hatzfeld M., Keil R., Magin T.M. (2017). Desmosomes and Intermediate Filaments: Their Consequences for Tissue Mechanics. Cold Spring Harb. Perspect. Biol..

