# Supplementary material for: Unveiling the Binding between the Armadillo-Repeat Domain of Plakophilin 1 and the Intrinsically Disordered Transcriptional Repressor RYBP
Source: Biomolecules. 2024 May 7;14(5):561. doi: 10.3390/biom14050561 (PMC11117474; doi:10.3390/biom14050561)

Supplementary Material

Unveiling the binding between the armadillo-repeat domain of Plakophilin 1 and the intrinsically disordered transcriptional repressor RYBP

Salome Araujo-Abad, Bruno Rizzuti, Miguel Vidal, Olga Abian, María Esther Fárez-Vidal, Adrian Velazquez-Campoy, Camino de Juan Romero and José L. Neira

FIGURE S1: **IF images of RYBP and PKP1.** RYBP (red), PKP1 (green) and DAPI (blue) in the two used cell lines. Scale bar: 10 μm.

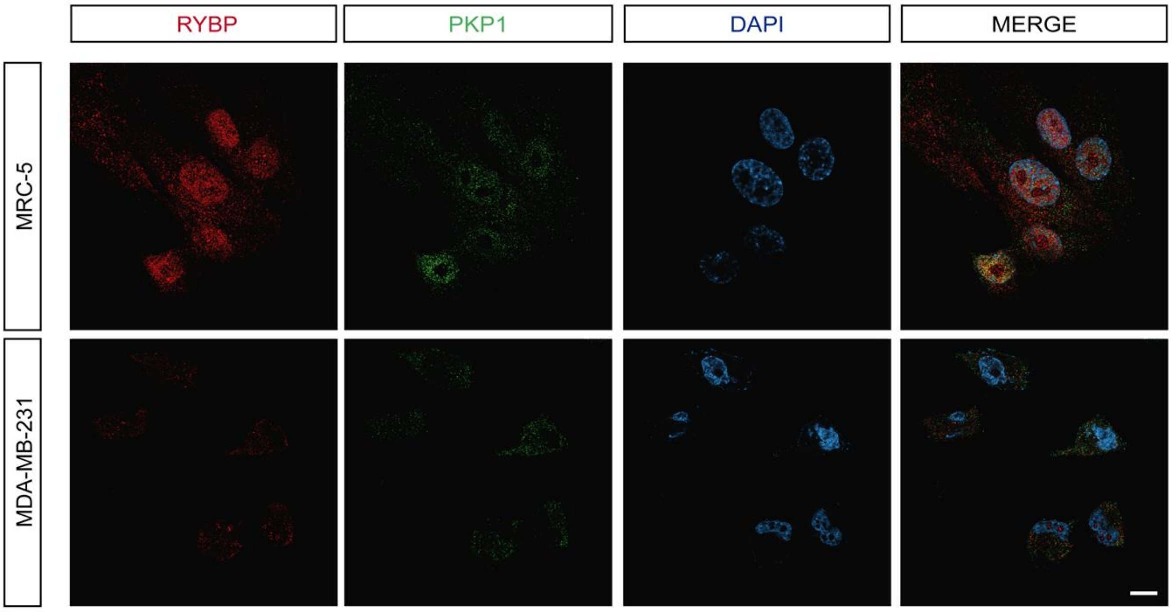

FIGURE S2: **Magnification of RYBP and PKP1 IF images.** RYBP (red), PKP1 (green), and DAPI (blue) in the two used cell lines. Scale bar: 10 μm.

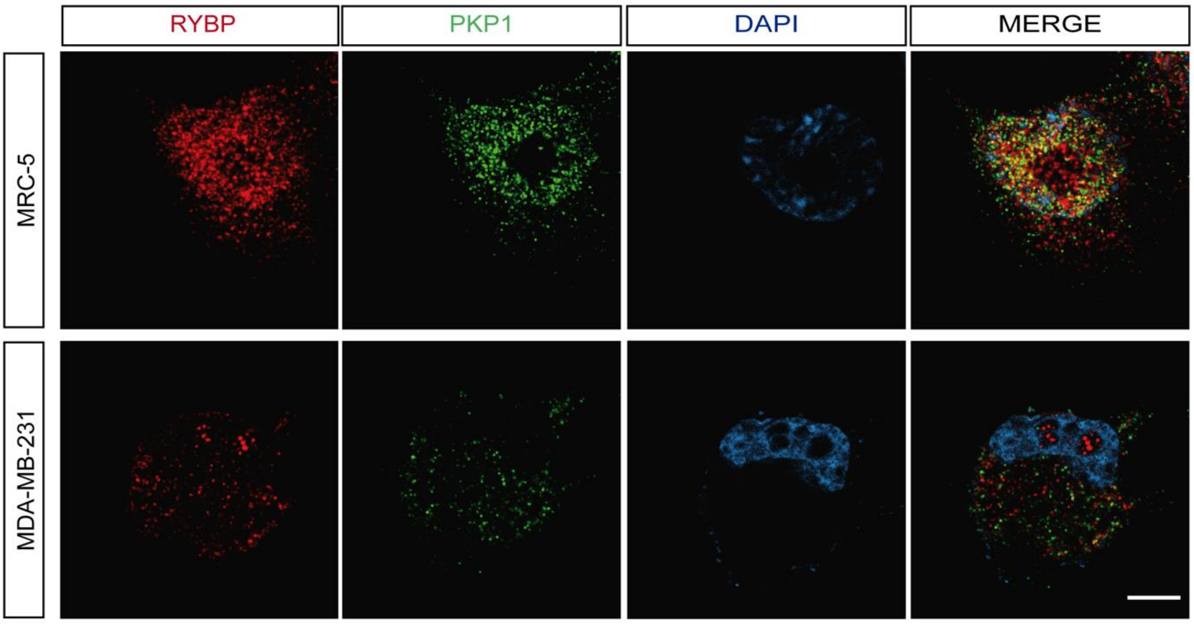

FIGURE S3: **PLAs of RYBP and PKP1.** PLA was performed in MRC-5 and MDA-MB-231 cells. A representative experiment is shown (n = 5). Scale bar: 10 µm

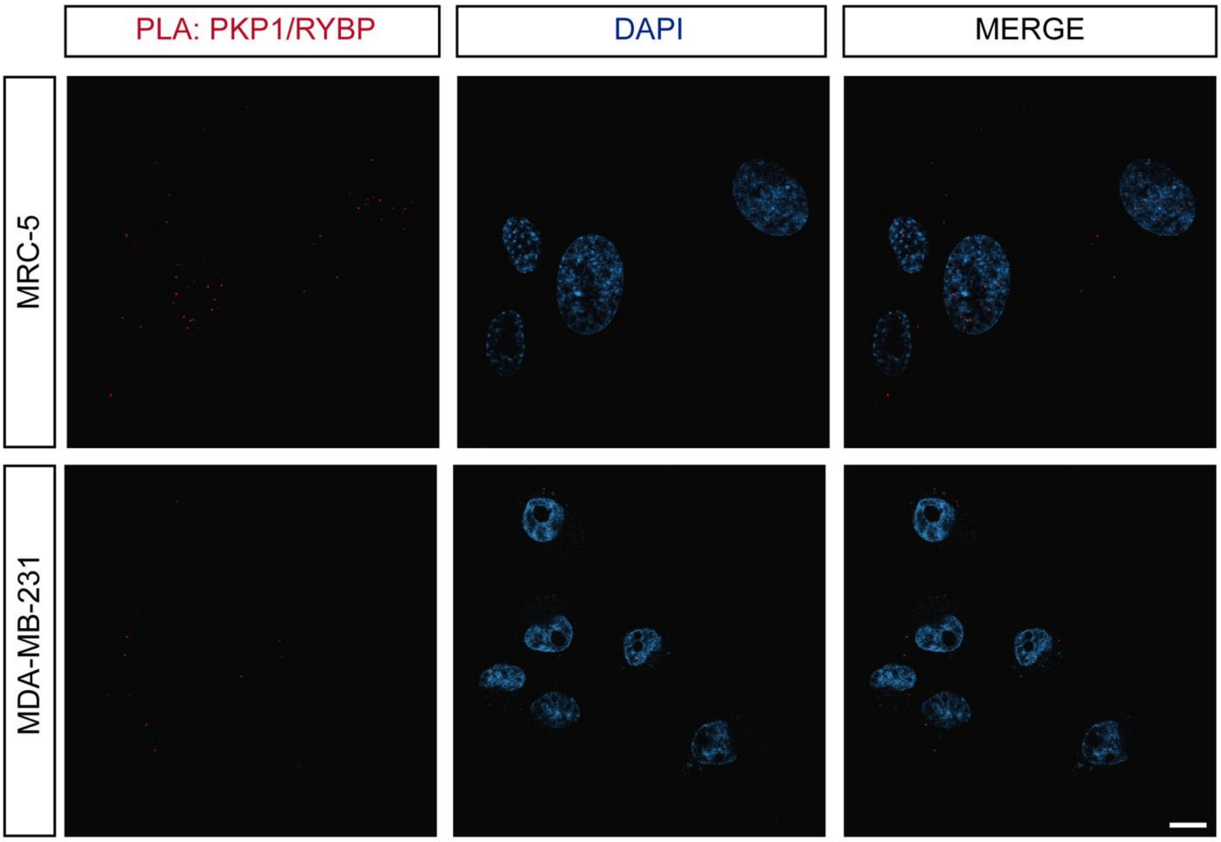

FIGURE S4: **In silico experiments of the complex between RYBP and ARM-PKP1.** (A) Binding locations of the fragments of RYBP on the surface ARM-PKP1 found in molecular docking simulations. Columns from left to right: fragment 20–55 (zinc-finger domain), 50–85 (N-term helix and NLS), and 145–180 (C-terminal β-hairpin). Rows from top to bottom: predictions of HawkDock (cyan), GRAMM (purple), GalaxyDock (orange). (B) Location of the basic binding patch on the inner surface of ARM-PKP1, and aromatic residues involved in the binding. In all cases ARM-PKP1 is shown with the innermost regions and the basic patch in front (see also Figure S5).

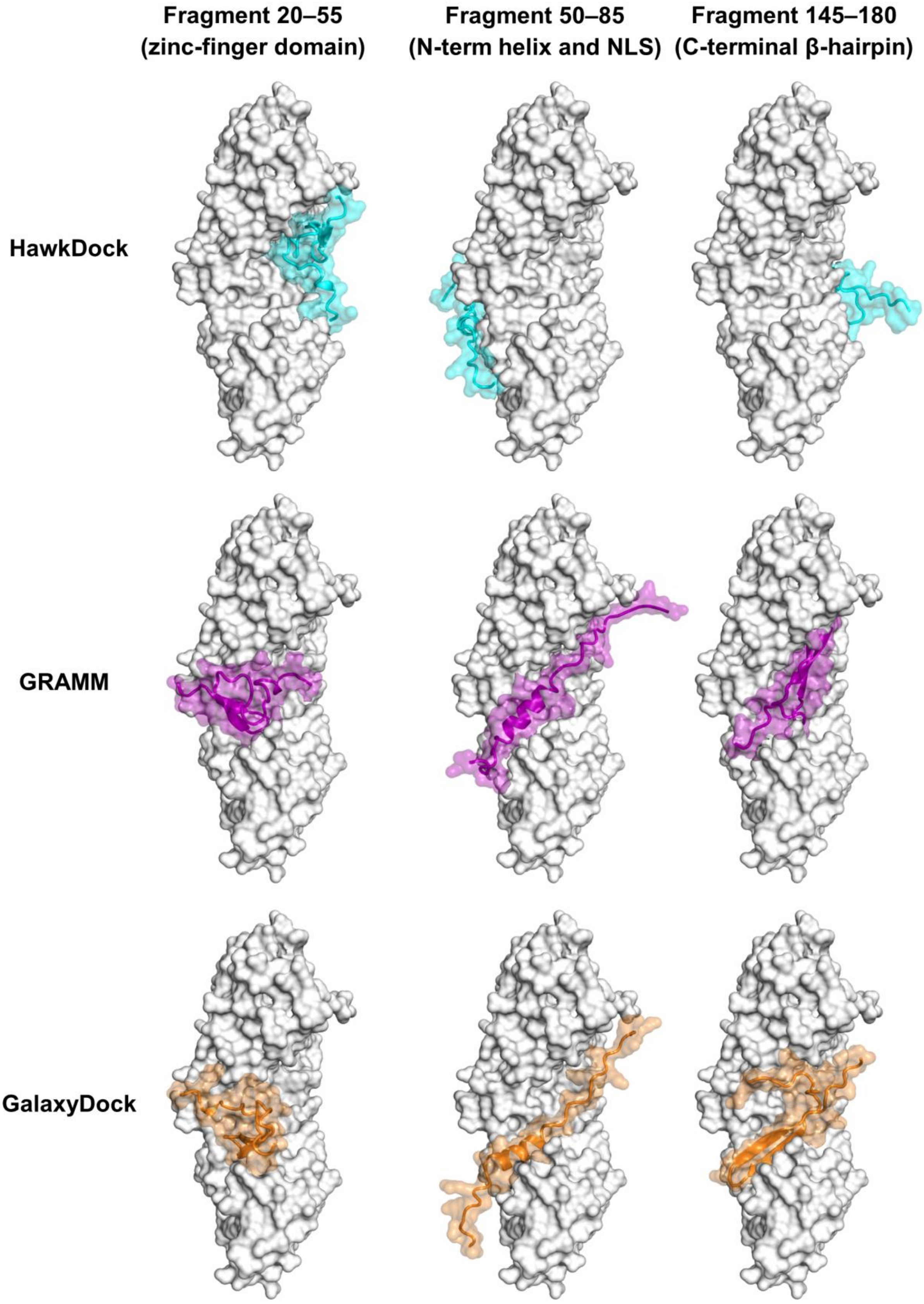

FIGURE S5: **Basic patch on the surface of ARM-PKP1 and key aromatic residues involved in the binding.** The protein is shown (left) with the innermost regions and the basic patch in front, and (right) rotated by 90°. The figure was colored by residue type: hydrophobic residues in black; basic residues in red; and acidic residues in blue.

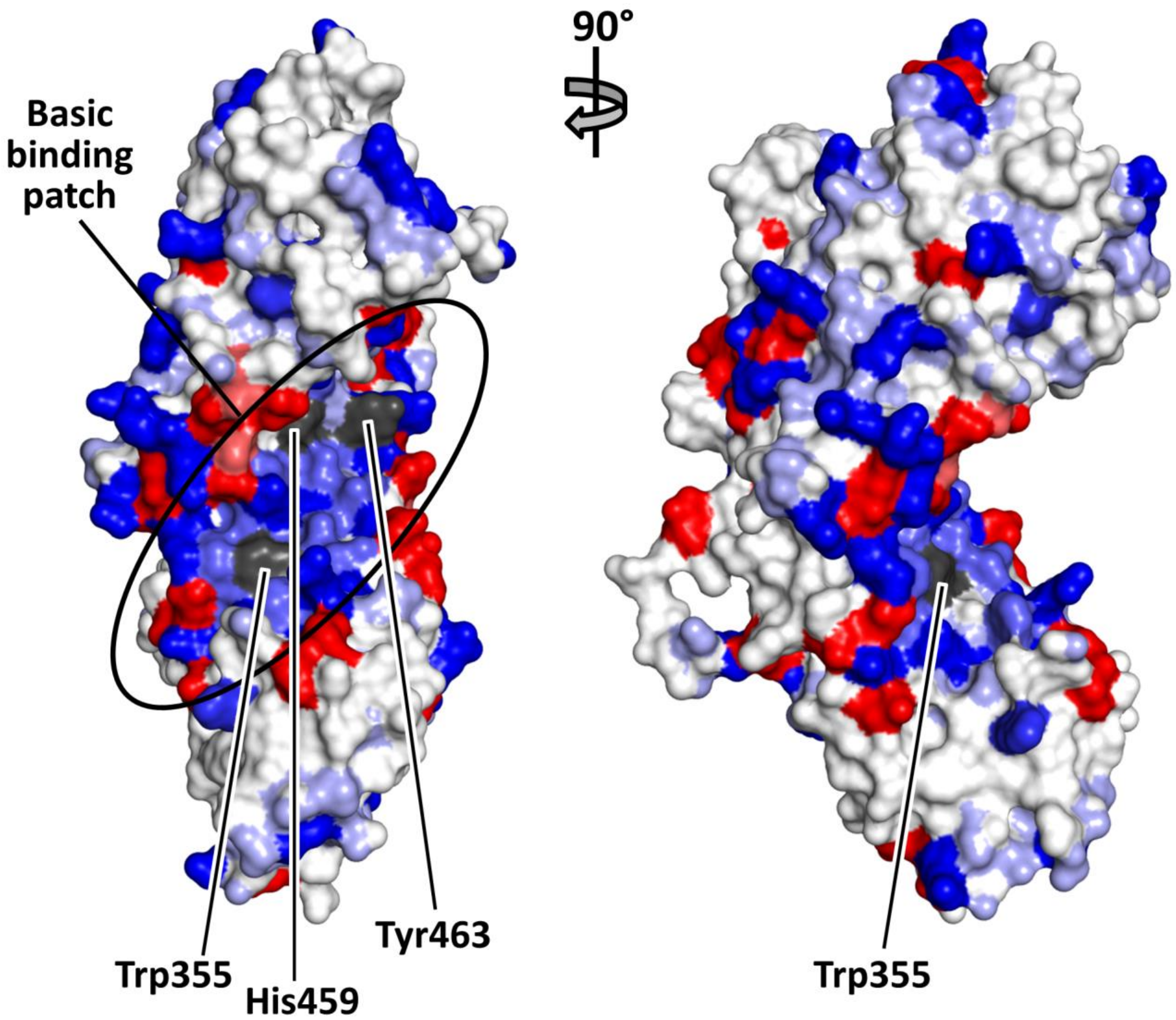

Supplement: Supplementary file 1 [file biomolecules-14-00561-s001.zip › biomolecules-2984234-supplementary.pdf]
